# Overexpression of Rice *Rab7* Gene Improves Drought and Heat Tolerance and Increases Grain Yield in Rice (*Oryza sativa* L.)

**DOI:** 10.3390/genes10010056

**Published:** 2019-01-17

**Authors:** Mohamed A. El-Esawi, Aisha A. Alayafi

**Affiliations:** 1 Botany Department, Faculty of Science, Tanta University, Tanta 31527, Egypt; 2 Biological Sciences Department, Faculty of Science, University of Jeddah, Jeddah 21577, Saudi Arabia; aal_shareaf@hotmail.com

**Keywords:** transgenic rice, *OsRab7*, drought and heat stress, antioxidants, gene expression

## Abstract

Rab family proteins play a crucial role in plant developmental processes and tolerance to environmental stresses. The current study investigated whether rice *Rab7* (*OsRab7*) overexpression could improve rice tolerance to drought and heat stress conditions. The *OsRab7* gene was cloned and transformed into rice plants. The survival rate, relative water content, chlorophyll content, gas-exchange characteristics, soluble protein content, soluble sugar content, proline content, and activities of antioxidant enzymes (CAT, SOD, APX, POD) of the transgenic rice lines were significantly higher than that of the wild-type. In contrast, the levels of hydrogen peroxide, electrolyte leakage, and malondialdehyde of the transgenic lines were significantly reduced when compared to wild-type. Furthermore, the expression of four genes encoding reactive oxygen species (ROS)-scavenging enzymes (*OsCATA*, *OsCATB*, *OsAPX2*, *OsSOD-Cu/Zn*) and eight genes conferring abiotic stress tolerance (*OsLEA3*, *OsRD29A*, *OsSNAC1*, *OsSNAC2*, *OsDREB2A*, *OsDREB2B*, *OsRAB16A*, *OsRAB16C*) was significantly up-regulated in the transformed rice lines as compared to their expression in wild-type. *OsRab7* overexpression also increased grain yield in rice. Taken together, the current study indicates that the *OsRab7* gene improves grain yield and enhances drought and heat tolerance in transgenic rice by modulating osmolytes, antioxidants and abiotic stress-responsive genes expression. Therefore, *OsRab7* gene could be exploited as a promising candidate for improving rice grain yield and stress tolerance.

## 1. Introduction

Drought and heat stresses are major abiotic factors that limit crops growth and productivity worldwide, with devastating agro-economic impacts [1]. Drought stress is frequently associated with heat stress [2,3]. Those abiotic factors influence different physiological processes in plants, causing the generation of high levels of reactive oxygen species (ROS) that have negative impacts on plant antioxidant systems and biological macromolecules [4]. As a result, plants evolve different homeostatic strategies, such as physiological, biochemical, and transcriptional responses to combat the adverse abiotic stress conditions [5,6,7,8,9]. These strategies include activation of antioxidant systems and modulation of compatible solutes and proteins mediating stress tolerance pathways. The transgenic approach is getting increasingly important for enhancing abiotic stress tolerance as well as developing abiotic stress-tolerant varieties [1]. Small GTP-binding proteins are widespread in eukaryotic cells and mediate several cellular processes, such as vesicular transport, cell proliferation, and signal transduction [10,11]. Those proteins comprise five important families (Rab, Rho, Ras, Ran, and Arf/Sar) [12,13]. Rab family proteins demonstrated crucial roles in diverse plant developmental processes, tolerance to environmental stresses, membrane organization, vesicle formation, and intracellular trafficking pathways [14]. Pereira-Leal and Seabra [15] described several Rab proteins in the *Arabidopsis* genome. Among those Rab proteins, *Rab7* demonstrated a high potential in enhancing plant tolerance to abiotic stresses. Mazel et al. [16] reported that *Rab7*-related proteins in *Arabidopsis* are localized on the vacuolar membrane and mediate the fusion of vesicle with vacuole. However, *Rab7*-related proteins in soybean were expressed in tonoplasts and endosomes, suggesting that *Rab7* multivesicular bodies play a regulatory role in endocytic pathways. Furthermore, various Rab proteins-encoding cDNAs and genes have been cloned from diverse plants. The functions of such genes have also been studied. For instance, *OsRab5a* gene, cloned from *Oryza sativa* L., played a crucial role in nutrient uptake in roots, early endosome transport, endosperm storage protein trafficking, and endomembrane organization [17,18,19]. *AtRabG3e* overexpression in *Arabidopsis* also enhanced sodium sequestration in vacuoles, and thereby augmented plant tolerance to salt and osmotic stresses [16]. *Pennisetum glaucum Rab7* overexpression augmented tobacco tolerance to salt and osmotic stresses [20]. Moreover, *RabG3b* gene promoted cell death during the plant senescence process and pathogen infection [21]. *OsRab7B3* overexpression conferred enhanced leaf senescence in rice [11]. Overexpression of rice *Rab7* (*OsRab7*) also improved salt tolerance in rice [22]. However, the functional role of *OsRab7* in mediating other important abiotic stresses has not been reported yet.

Rice (*Oryza sativa* L.) is one of the most important food crops worldwide and it requires huge quantities of water during growth cycle [23]. Drought and heat stresses negatively influence crops growth and yield [23,24,25]. Several studies have reported the use of a potential transgenic approach in enhancing drought and heat stress tolerance in rice [23,26,27,28,29,30,31,32]. Wu et al. [23] reported enhanced drought and heat tolerance in the rice over-expressing *OsWRKY11* transcription factor. Moreover, enhanced drought tolerance levels have been reported in rice overexpressing different genes, such as *OsHsfA7* [26], *LEA* [27], *OsNAC14* [28], *OsDRAP1* [29], *XERICO* [30], *OsLG3* [31], or *OsHSP50.2* [32]. Overexpression of *DPB3-1* or *OsHTAS* also improved heat tolerance in rice [33,34]. However, developing more transgenic stress-tolerant rice varieties is currently essential for use in breeding strategies to overcome adverse water deficit and heat stress effects and meet the increasing worldwide population demands. When considering the aforementioned remarks, the current study aimed to investigate whether the overexpression of *OsRab7* gene, cloned from *O. sativa* L., could enhance drought and heat tolerance and improve grain yield in transgenic rice plants. In addition to the grain yield parameters, several physiological, biochemical, and transcriptional analyses were conducted to compare between the transgenics and wild type plants.

## 2. Materials and Methods

### 2.1. Plant Material and Growth Conditions

Rice (*O. sativa* ssp. *Japonica* cv. Giza 177) genotype was obtained from the Agricultural Research Center in Egypt and was used to generate transgenic lines in the present study. The wild-type and transgenic rice seeds were surface-sterilized and germinated on a wet paper for 4 days. The uniform seedlings were then transplanted into plastic pots containing mixed soil of sand, peat, and perlite (1:1:1, v/v/v) and grown in a growth chamber under conditions of 26/20 °C (day/night), 16/8 h (light/dark), and humidity of 70%. The plants were irrigated daily with sterile distilled water. 

### 2.2. Plasmid Construction and Rice Transformation

Total RNA was isolated from three-week-old rice seedlings using RNeasy Plant Mini kit (Qiagen, Hilden, Germany), followed by the removal of contaminating DNA using RNase-Free DNase Set (Qiagen). Complementary DNA (cDNA) synthesis was carried out using Reverse Transcription kit (Qiagen). The full length cDNA of *OsRab7* was then amplified using the primer pair designed by Peng et al. [22]. cDNA *OsRab7* was then digested with *Bam*HI and ligated to the modified binary vector plasmid pCU, as previously described [22,35]. The resulting constructs, *pOsRab7*, were introduced into *Agrobacterium tumefaciens* EHA105, which was then used for the transformation of rice cultivar Giza 117 (*Oryza sativa* L. ssp. *japonica*) following the *Agrobacterium*-mediated transformation method [36]. Transgenic plant seeds were selected on Murashige and Skoog (MS) media containing 40 mg L^−1^ hygromycin.

### 2.3. Molecular Analysis of Transgenic Plants and Transgene Expression

Putative transgenic plants survived on MS medium containing 40 mg L^−1^ hygromycin were further verified by polymerase chain reaction (PCR) amplification of hygromycin resistant gene (hygromycin phosphotransferase, *hpt*), as previously reported by Peng et al. [22]. Seedlings were then transplanted into soil to obtain T_3_ seeds, as this generation reveals more homozygosity and stable gene expression. The transgenic *OsRab7* gene cope number was examined by southern blot analysis. In brief, genomic DNA was isolated from the leaves of the wild-type and T_3_ transgenic rice lines. Isolated DNA was then digested, electrophoresed on 0.8% (*w*/*v*) agarose gel, transferred onto a Hybond-N^+^ nylon membrane (Roche), and hybridized using the hygromycin-resistant gene.

*OsRab7* gene overexpression was also examined in the wild-type and T_3_ transgenic rice lines using quantitative real-time PCR (qRT-PCR) analysis. As described above, RNA isolation and cDNA synthesis from wild-type and T_3_ transgenic lines were conducted. qRT-PCR analysis was conducted in triplicates (three biological replicates and three technical replicates), according to the manufacturer’s procedures of QuantiTect SYBR Green PCR kit (Qiagen). PCR reactions and amplification conditions were conducted, as previously reported by Peng et al. [22]. Specific primers previously designed for *OsRab7* [22] was used for amplification. *Actin* was used as a housekeeping gene [22]. *OsRab7* expression was quantified using 2^−ΔΔCt^ method.

### 2.4. Drought and Heat Stress Treatments

Wild-type and 3 T_3_ homozygous transgenic rice lines exhibiting the highest *OsRab7* expression level (OE-3, OE-4, OE-6) were used for drought and heat stress treatment experiments. The wild-type and the three T_3_ transgenic rice lines were germinated on a wet paper for four days. The healthy uniform seedlings were then transplanted into plastic pots containing sandy soil and grown in a growth chamber with daily irrigation with sterile distilled water for three weeks under conditions of 26/20 °C (day/night), 16/8 h (light/dark), and humidity of 70%. The 25-day-old plants were divided into three groups used for the following treatments; (i) control at 26/20 °C (day/night) with irrigation every day; (ii) drought stress at 26/20 °C (day/night) without irrigation; and, (iii) heat stress, as recommended by Aghamolki [24] at 40/32 °C (day/night) with irrigation every day. All of the treatments lasted for 10 days at which plants were collected for use in the subsequent physiological, biochemical and transcriptional analyses. Following the 10-day stress treatments, 10-day recovery was applied to calculate the survival rate. The above experiments were repeated thrice.

To evaluate the yield components of the wild-type and transgenic rice plants under normal, drought, and heat stress conditions, the wild-type and the 3 T_3_ homozygous transgenic rice lines were transplanted into 1-m-deep containers containing natural paddy soil located in the greenhouse at 26/20 °C (day/night). The containers were arranged in a completely randomized design, and the plants were watered daily. Approximately 10–15 days before the panicle heading stage, the following treatments were applied: (i) control, 26/20 °C (day/night) with irrigation; (ii) drought stress, 26/20 °C (day/night) without irrigation; and, (iii) heat stress, 40/32 °C (day/night) with irrigation. All of the treatments lasted for 25 days, followed by growing under normal growth conditions till harvesting. When the grains ripened, the following yield parameters were scored; panicle length (cm), number of spikelets per panicle, total number of spikelets per hill, number of filled grains per hill, filling rate (%), and total grain weight (g).

### 2.5. Determination of Growth Traits and Relative Water Content

Plant height, root fresh weight and shoot fresh weight of the collected plants were determined. Leaf relative water content (RWC) was estimated according to the method described by Yamasaki and Dillenburg [37].

### 2.6. Estimation of Oxidative Stress Biomarker Levels

Electrolyte leakage (*EL*) was estimated using the methodology that was described by Dionisio-Sese and Tobita [38]. Hydrogen peroxide (H_2_O_2_) was estimated by homogenizing fresh tissue (50 mg) in TCA (0.5 mL, 0.1%), followed by centrifuging the resulting homogenate and calculating H_2_O_2_ content following the methodology of Velikova et al. [39]. Malondialdehyde (MDA) content was estimated using the method reported by Rao and Sresty [40].

### 2.7. Estimation of Gas-Exchange Characteristics

The transpiration rate (*E*), net photosynthesis rate (*P_n_*), and stomatal conductance (*g_s_*) were estimated in leaves at 10:00 a.m., as reported by Holá et al. [41], using a portable gas-exchange system *LCpro*+ (ADC BioScientific Ltd., Hoddesdon, UK).

### 2.8. Determination of Soluble Proteins, Soluble Sugars, Proline and Chlorophyll Contents

Fresh leaves were homogenized in 1 mL of 100 mM Tris buffer (pH 8.0), followed by centrifugation at 20,000× *g* and 4 °C for 12 min. Soluble proteins in leaves were quantified following Bradford methodology [42]. Soluble sugars were quantified, as reported by Dey [43]. Proline content was estimated using the method of Bates et al. [44] by recording the absorbance at 520 nm.

To estimate chlorophyll content, leaf disks were homogenized and extracted in 80% acetone, and absorpance was spectrophotometrically taken at 645 and 663 nm [7].

### 2.9. Antioxidant Enzyme Assays

Fresh leafy samples were ground in liquid N_2_, standardized using 0.05 M phosphate buffer (pH 7.8) and centrifuged at 14,000× *g* for 8 min at 5 °C. Supernatant was then used for measuring antioxidant enzymes activities. Catalase (CAT) activity was determined, as reported by Aebi [45], and absorbance was spectrophotometrically recorded at 240 nm. Peroxidase (POD) and superoxide dismutase (SOD) activities were measured following the methodology of Zhang [46]. Ascorbate peroxidase (APX) activity was measured following the methodology that was reported by Nakano and Asada [47], and absorbance was spectrophotometrically measured at 265 nm.

### 2.10. Stress-Related Genes Expression Analysis

Quantitative real-time PCR was carried out to measure the expression levels of four antioxidants genes (*OsCATA*, *OsCATB*, *OsAPX2*, *OsSOD-Cu/Zn*) and eight genes conferring abiotic stress tolerance (*OsLEA3*, *OsRD29A*, *OsSNAC1*, *OsSNAC2*, *OsDREB2A*, *OsDREB2B*, *OsRAB16A*, *OsRAB16C*) in the wild-type and the three T_3_ transgenic rice lines subjected to normal, drought, or heat stress conditions. RNA isolation and cDNA synthesis from the plant tissues were performed, as described above. qRT-PCR analysis was carried out in triplicates (three biological repeats and three technical repeats), following the manufacturer’s procedures of QuantiTect SYBR Green PCR kit (Qiagen). PCR reactions and amplification conditions for the four antioxidants genes and the eight genes conferring abiotic stress tolerance were conducted, as previously reported by Vighi et al. [48] and Cai et al. [49], respectively. Specific primers that were previously designed for the four antioxidants genes [48] and the eight genes conferring abiotic stress tolerance [49,50] were used for amplification. *UBQ10* was used as an internal reference gene [48]. Genes expression levels were quantified following the 2^−ΔΔCt^ method [51].

### 2.11. Statistical Analysis

One-way analysis of variance (ANOVA) was performed for the collected data using SPSS version 16.0. Values are means ±SE (*n* = 3), and differ significantly at *p* ≤ 0.05.

## 3. Results and Discussion

### 3.1. Transformation and Molecular Analysis of Transgenic Rice Lines

**Figure 1:**
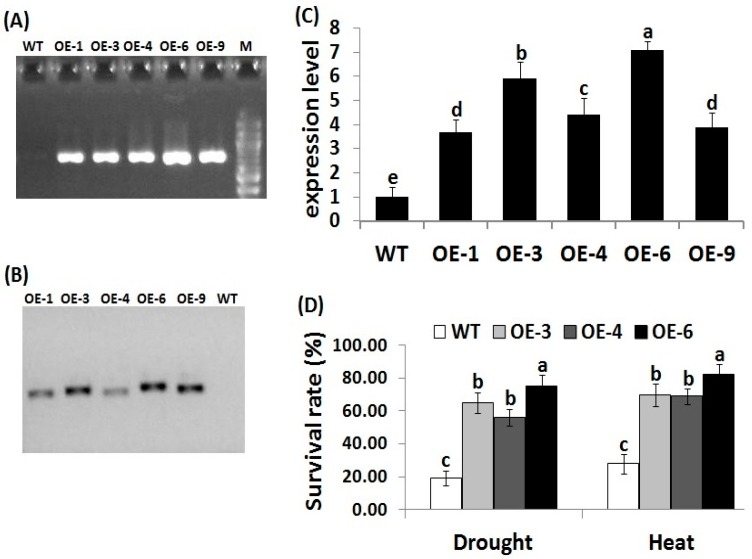
Molecular analysis of the transgenic rice lines. (**A**) PCR amplification of 750 bp of *hpt* hygromycin resistant gene in the wild-type (WT) and the 5 transgenic (OE) lines. (**B**) Southern blot analysis of the digested genomic DNA from the WT and the 5 transgenic lines. (**C**) Relative expression of *OsRab7* in transgenic lines using qRT-PCR analysis. (**D**) Survival rate of the WT and transgenic lines following 10-day recovery after drought and heat treatments. M, 100 bp DNA ladder. Different letters above the columns indicate significant differences between lines (*p* ≤ 0.05).

Genetic transformation technology has become a potential tool to enhance crop stress tolerance and accelerate breeding strategies. To investigate whether *OsRab7* overexpression enhances drought and heat stress tolerance in rice, transgenic rice lines overexpressing *OsRab7* were generated using *Agrobacterium*-mediated transformation. Sixteen independent rice transgenic lines were obtained. Five positive transgenic lines designated OE-1, OE-3, OE-4, OE-6, and OE-9 had sufficient seeds and were selected and further verified by PCR amplification of hygromycin resistant gene. The expected fragments of a size of 750 bp were amplified in the five transgenic rice lines, but not in the wild-type plants (Figure 1A). Southern blot analysis confirmed the successful integration of the transformed *OsRab7* gene into the genomes of lines OE-1, OE-3, OE-4, OE-6, and OE-9, with one single copy present in each transgenic line (Figure 1B). *OsRab7* transcription levels were also detected in the five T_3_ rice transformants (OE-1, OE-3, OE-4, OE-6, and OE-9) using qRT-PCR (Figure 1C). The three T_3_ transgenic rice lines (OE-3, OE-4, OE-6) exhibited the highest *OsRab7* expression levels and they were therefore selected for subsequent physiological, biochemical, and transcriptional analyses.

### 3.2. OsRab7 Overexpression in Rice Enhances Survival Rate, Plant Growth, and Relative Water Content under Drought and Heat Stress Conditions

Following drought and heat stress treatments, a 10-day growth recovery was conducted. The survival rate of the transgenic rice lines (OE-3, OE-4, OE-6) was significantly higher than that of the wild-type (Figure 1D). Phenotypic traits serve as indicators for the identification of stress tolerance in plants. Leaf relative water content is also an important indicator of water status balance in plants [52,53]. Decrease in relative water content causes osmotic stress, which in turn affects plant growth [54]. In the present study, under normal conditions, no obvious differences were recorded in plant height, root and shoot fresh weight, or relative water content between the wild-type and the transgenic lines (Table 1). Under drought and heat stress conditions, decreases in the growth traits and relative water content were recorded for the wild-type and transgenic lines as compared to normal conditions. However, transgenic lines exhibited significantly higher growth parameters and relative water content when compared to the wild-type under drought and heat stress conditions (Table 1). These results indicated that *OsRab7* overexpression in transgenic rice plants enhances their survival rate, growth, relative water content, and tolerance to drought and heat stresses.

### 3.3. OsRab7 Overexpression in Rice Reduces Oxidative Stress Biomarkers under Drought and Heat Stress Conditions

**Figure 2:**
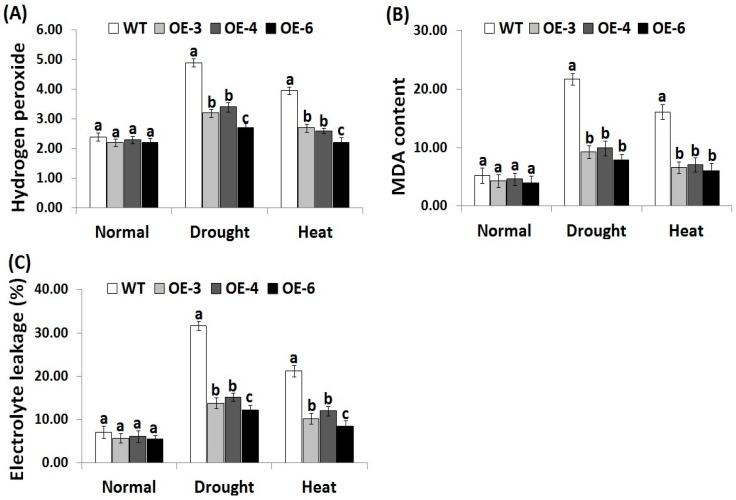
Hydrogen peroxide (H_2_O_2_, µmol g^−1^ FW) content (**A**), lipid peroxidation (malondialdehyde (MDA), µmol g^−1^ FW) level (**B**), and electrolyte leakage (**C**) of the wild-type and transgenic rice plants overexpressing *OsRab7* subjected to normal, drought and heat stress conditions. Data are means ± SE (*n* = 3). Different letters above the columns indicate significant differences between rice lines (*p* ≤ 0.05). SE: Standard error.

Reactive oxygen species have toxic impacts on plants [4]. Electrolyte leakage is a key indicator for the plant cell membrane damage [55]. MDA also indicates lipid peroxidation end products damage caused by free radicals [56]. Therefore, developing more stress-tolerant varieties scavenging ROS is importantly needed. In order to investigate whether the transgenic rice lines overexpressing *OsRab7* could scavenge ROS, oxidative stress biomarkers, such as H_2_O_2_, MDA, and *EL* were estimated in the wild-type and the three transgenic lines under drought and heat stress conditions. Under normal conditions, there were no significant differences in H_2_O_2_, MDA, and *EL* levels between the wild-type and the three transgenic lines (Figure 2A–C). Under drought and heat stress conditions, increases in H_2_O_2_, MDA, and *EL* levels were recorded for the wild-type and transgenic lines as compared to normal circumstances. However, transgenic lines exhibited significantly lower levels of H_2_O_2_, MDA, and *EL* as compared to the wild-type under drought and heat stress conditions (Figure 2A–C). These results indicate that ROS accumulation in transgenic lines overexpressing *OsRab7* is much lower than that of the wild-type. Thus, *OsRab7* overexpression in transgenic rice plants counteract the toxic ROS effects and reduce the oxidative damage, conferring greater tolerance to drought and heat stresses.

### 3.4. OsRab7 Overexpression in Rice Improves Gas-Exchange Characteristics Under Drought and Heat Stress Conditions

**Figure 3:**
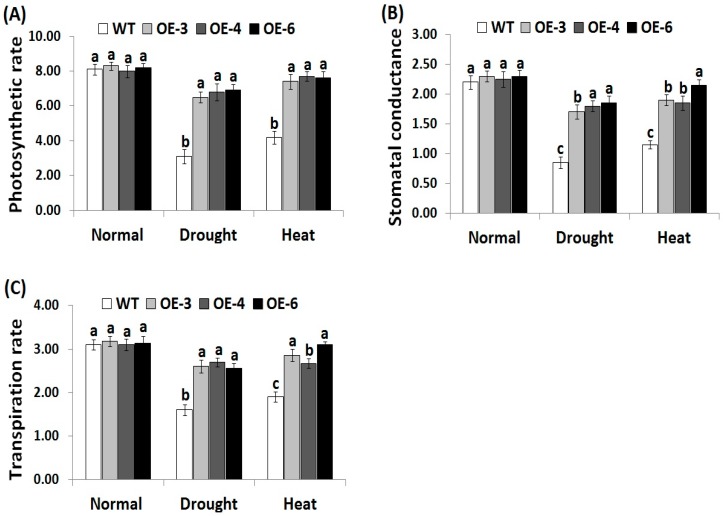
Photosynthesis rate (*P_n_*, μmol m^2^s^−1^) (**A**), stomatal conductance (*g_s_*, mol m^2^s^−1^) (**B**), and transpiration rate (*E*, mmol m^2^s^−1^) (**C**) of the wild-type and transgenic rice plants overexpressing *OsRab7* subjected to normal, drought, and heat stress conditions. Data are means ± SE (*n* = 3). Different letters above the columns indicate significant differences (*p* ≤ 0.05).

Gas-exchange parameters are significantly affected by the adverse effects of abiotic stresses. To evaluate whether *OsRab7* overexpression in rice could improve gas-exchange parameters under abiotic stress, we estimated photosynthesis rate, stomatal conductance, and transpiration rate in the wild-type and the three *OsRab7*-overexpressing rice lines under drought and heat stress conditions. Under normal growth conditions, the transgenic rice lines did not reveal any significant difference in gas-exchange attributes when compared to the wild-type (Figure 3A–C). Under drought and heat stress conditions, decreases in gas-exchange parameters were recorded for the wild-type and transgenic lines compared to the normal growth conditions. However, transgenic lines revealed significantly higher levels of gas-exchange attributes as compared to the wild-type under drought and heat stress conditions (Figure 3A–C), indicating that the *OsRab7*-overexpressing rice lines exhibited greater tolerance to drought and heat stress effects through enhancing the relative water content and gas-exchange characteristics.

### 3.5. OsRab7 Overexpression in Rice Improves Osmolytes and Chlorophyll Content under Drought and Heat Stress Conditions

**Figure 4:**
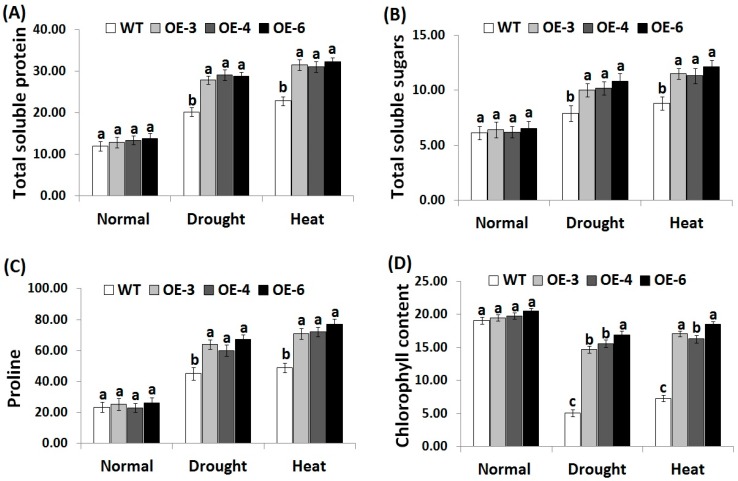
Contents of total soluble protein (mg g^−1^ FW) (**A**), soluble sugars (mg g^−1^ FW) (**B**), proline (µg g^−1^ FW) (**C**) and chlorophyll (mg g^−1^ FW) (**D**) of the wild-type and transgenic rice plants overexpressing *OsRab7* subjected to normal, drought and heat stress conditions. Data are means ± SE (*n* = 3). Different letters above the columns indicate significant differences between lines (*p* ≤ 0.05).

Osmotic adjustment represents one of the most important features of abiotic stress tolerance in plants, while soluble proteins, soluble sugars, proline, and other compatible solutes serve as osmoprotectants under stress conditions [57,58]. Soluble proteins and sugars also protect plant cells from dehydration and stabilize and protect the biological molecules function [59]. In the present study, the contents of soluble proteins, soluble sugars, proline, and chlorophyll were estimated in the wild-type and the three *OsRab7*-overexpressing rice lines under drought and heat stress conditions in order to understand the transgenic lines osmoregulation ability (Figure 4A–D). Non-significant slight differences in the contents of soluble proteins, sugars, proline, and chlorophyll were recorded between the wild-type and transgenic lines under normal growth conditions. By contrast, under drought and heat stress conditions, a remarkable increase in the contents of soluble proteins, sugars, proline, and chlorophyll was recorded for the wild-type and transgenic lines compared to normal growth conditions (Figure 4A–D). Nevertheless, transgenic lines revealed significantly higher contents of soluble proteins, sugars, proline and chlorophyll as compared to the wild-type under drought and heat stress conditions. These results reveal that *OsRab7* overexpression enhanced the contents of compatible solutes and chlorophyll and promoted the osmoregulation ability in the transgenic rice lines, resulting in improved tolerance to the drought and heat stresses.

### 3.6. OsRab7 Overexpression in Rice Induces Antioxidant Enzyme Activities under Drought and Heat Stress Conditions

**Figure 5:**
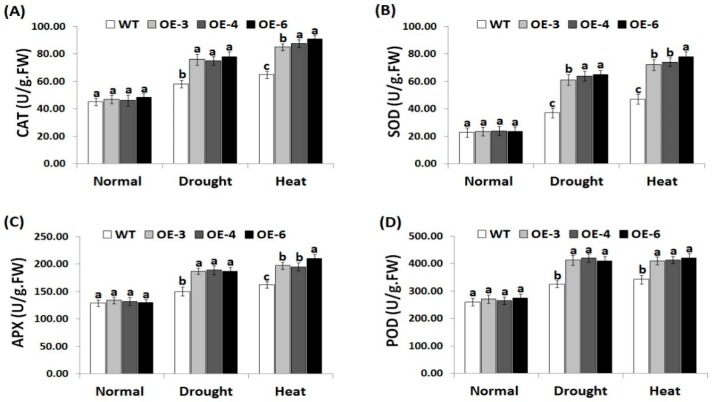
Activities of catalase (CAT) (**A**), superoxide dismutase (SOD) (**B**), ascorbate peroxidase (APX) (**C**), and peroxidase (POD) (**D**) in the wild-type and transgenic rice plants overexpressing *OsRab7* subjected to normal, drought, and heat stress conditions. Data are means ± SE (*n* = 3). Different letters above the columns indicate significant differences between rice lines (*p* ≤ 0.05).

Antioxidant enzymes play a key role in scavenging ROS and improving plant tolerance to environmental stresses [8]. Therefore, we estimated the activities of CAT, SOD, APX, and POD in the wild-type and *OsRab7*-overexpressing rice plants (Figure 5A–D). Under normal growth conditions, there were no significant differences in the antioxidant enzymes activities between the wild-type and the three transgenic lines (Figure 5A–D). By contrast, under drought and heat stress conditions, remarkable increases in the antioxidant enzymes activities were recorded for the wild-type and transgenic lines compared to normal growth conditions. However, transgenic lines revealed significantly higher antioxidant enzymes activities when compared to the wild-type under drought and heat stress conditions. As a result, *OsRab7* overexpression significantly induces the antioxidant enzymes activities of transgenic rice lines to counteract the toxic impacts of ROS, resulting in reduced oxidative damage in plant cells.

### 3.7. OsRab7 Overexpression in Rice Induces Abiotic Stress-Related Genes Expression under Drought and Heat Stress Conditions

**Figure 6:**
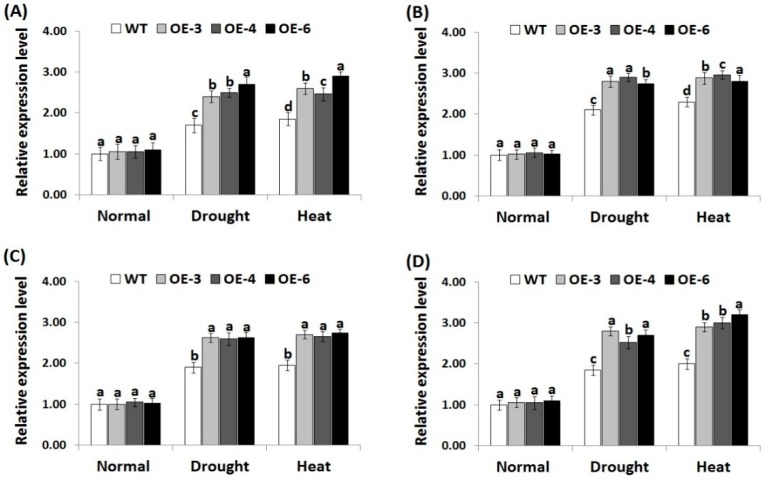
Expression levels of *OsCATA* (**A**), *OsCATB* (**B**), *OsAPX2* (**C**), and *OsSOD-Cu/Zn* (**D**) genes in the wild-type and transgenic rice plants overexpressing *OsRab7* subjected to normal, drought, and heat stress conditions. Data are means ± SE (*n* = 3). Different letters above the columns indicate significant differences between rice lines (*p* ≤ 0.05).

**Figure 7:**
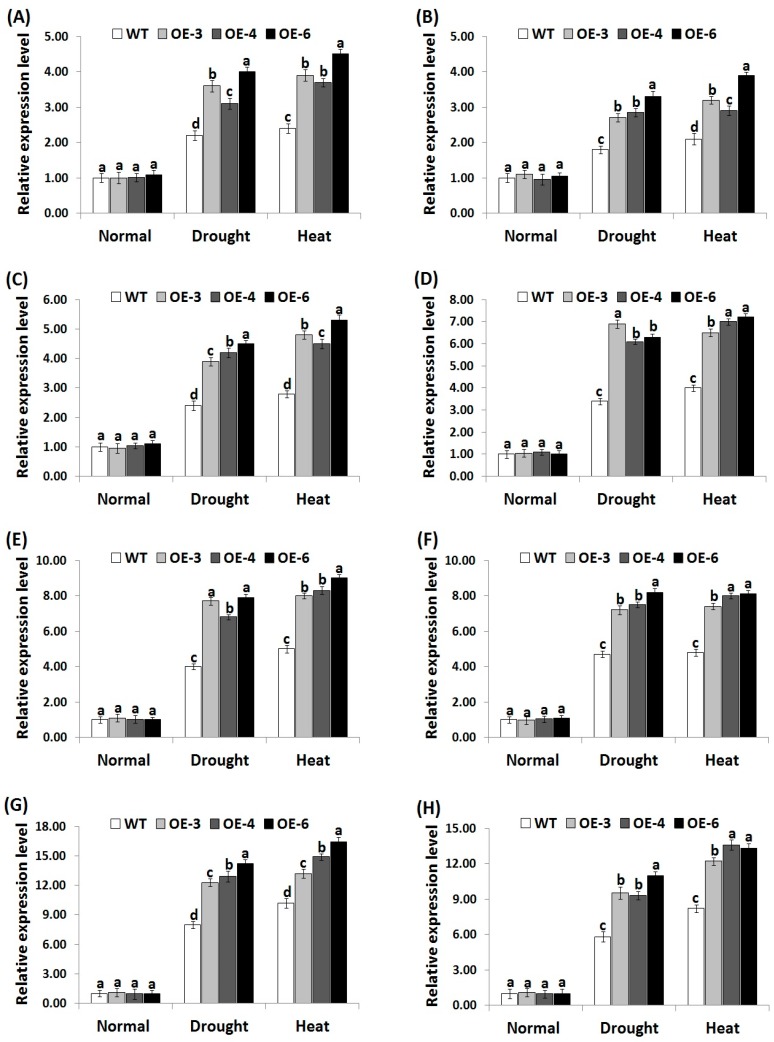
Expression levels of *OsLEA3* (**A**), *OsRD29A* (**B**), *OsSNAC1* (**C**), *OsSNAC2* (**D**), *OsDREB2A* (**E**), *OsDREB2B* (**F**), *OsRAB16A* (**G**), and *OsRAB16C* (**H**) genes in the wild-type and transgenic rice plants overexpressing *OsRab7* under normal, drought, and heat stress conditions. Data are means ± SE (*n* = 3). Different letters above columns indicate significant difference between rice lines (*p* ≤ 0.05).

In order to investigate the signaling regulatory role of *OsRab7* overexpression in stress tolerance mechanisms, we measured the expression level of four genes encoding ROS-scavenging enzymes (*OsCATA*, *OsCATB*, *OsAPX2*, *OsSOD-Cu/Zn*) and eight genes conferring abiotic stress tolerance (*OsLEA3*, *OsRD29A*, *OsSNAC1*, *OsSNAC2*, *OsDREB2A*, *OsDREB2B*, *OsRAB16A*, *OsRAB16C*) in the wild-type and the three transgenic rice lines under drought or heat stress conditions using qRT-PCR. Under normal growth conditions, non-significant slight differences in the expression of antioxidant genes and abiotic stress-related genes were recorded between the wild-type and transgenic lines (Figure 6A–D; Figure 7A–H). By contrast, under drought and heat stress conditions, a remarkable induction in the expression of all genes was recorded for the wild-type and transgenic lines as compared to normal growth conditions. Nevertheless, transgenic lines exhibited significantly higher expression levels of antioxidant genes (Figure 6A–D) and abiotic-stress related genes (Figure 7A–H) as compared to the wild-type under drought and heat stress conditions. These results indicate that *OsRab7* overexpression reduces oxidative damages via inducing ROS scavenging pathways and proteins that are involved in defence mechanisms, thereby improving drought and heat tolerance.

In consistent with the results of antioxidant enzyme assays, the expression level of the antioxidant genes and abiotic stress-related genes was significantly induced in transgenic rice lines under stress conditions. These results were in harmony with previous report [49], which indicated that rice overexpressing rat neuronal NO synthase (*nNOS*) exhibited much higher expression levels of antioxidant genes (*OsCATA*, *OsCATB*, *OsPOX1*) and abiotic stress-responsive genes (*OsLEA3*, *OsDREB2A*, *OsDREB2B*, *OsRD29A*, *OsSNAC1*, *OsSNAC2*) as compared to the wild-type under drought and salt stress conditions. Moreover, the results were in harmony with the previous findings of Yu et al. [50], who reported that rice plants overexpressing *OsEm1* exhibited higher expression levels of late embryogenesis abundant proteins (*OsLEA3*, *OsRAB16A*, *OsRAB16C*) involved in abiotic stress tolerance as compared to the wild-type under drought stress. The results are also in harmony with the previous reports that also indicated the key role of Rab proteins in enhancing plant tolerance to abiotic stress [16,22]. Rab7 proteins are involved in vesicle trafficking and localized in the tonoplast in Arabidopsis and rice [60,61,62]. Enhanced vesicle trafficking was reported in soybean plants overexpressing *Rab7* [63]. Peng et al. [22] also reported that *OsRab7* overexpression enhanced rice salt tolerance through enhancing vesicle trafficking. Moreover, previous studies reported that osmotic stress signaling transduction could be mediated by the intracellular vesicle trafficking, as demonstrated by the role of phosphatidylinositol signaling in vesicle trafficking and stress tolerance [64,65]. Therefore, together with the previously demonstrated role of *OsRab7* in inducing vesicle trafficking, the present study indicates that *OsRab7* overexpression might induce drought and heat tolerance via enhancing vesicle trafficking in rice.

### 3.8. OsRab7 Overexpression in Rice Increases Grain Yield under Drought and Heat Stress Conditions

Rice grain yield is severely influenced upon exposure to drought and heat stress. Therefore, it was important to evaluate the adverse impacts of drought and heat stress on grain yield of the wild-type and the three transgenic rice lines overexpressing *OsRab7* by recording various yield parameters, such as panicle length, number of spikelets per panicle, total number of spikelets per hill, number of filled grains per hill, filling rate, and total grain weight. The evaluation of these yield components showed that there were no obvious differences in the grain yield between the wild-type and the transgenic lines (Table 2). However, under drought and heat stress conditions, reductions in the grain yield parameters were recorded for the wild-type and transgenic lines as compared to normal growth conditions. Nevertheless, transgenic lines exhibited significantly lower decreases in grain yield when compared to the wild-type under drought and heat stress conditions (Table 2). For example, the transgenic lines had a higher filling rate than that of the wild type plants under drought and heat stress conditions, resulting in increases in the total grain weight. These results were in consistent with that of previous studies which reported higher rice grain yield in rice plants overexpressing *AP37* [66], *TIFY* [67], *OsNAC2* [68], and *OsNRT2.1* [69]. 

## 4. Conclusions

Drought and heat stress affects rice growth and yield. To improve rice tolerance to those adverse stresses, *OsRab7* was cloned and overexpressed in rice plants. The wild-type and three generated transgenic rice lines were included in stress treatments. The results showed that *OsRab7* overexpression improves rice tolerance to drought and heat stress by modulating gas-exchange attributes, osmolytes, photosynthesis, antioxidant machinery, and expression of genes that are involved in defence pathways. The present study also demonstrated that *OsRab7* overexpression increases grain yield in transgenic rice. Therefore, *OsRab7* gene could serve as a candidate for enhancing stress tolerance and grain yield in rice. Future work should explore the regulatory network and function of *OsRab7* in greater depth to unravel the mechanisms controlling stress tolerance in rice.

## Figures and Tables

**Table 1:** Growth, biomass, and relative water content of the WT and transgenic rice seedlings grown under normal, heat, and drought stress conditions.

Treatment	Line	Plant Height (cm)	Root Fresh Weight (mg)	Shoot Fresh Weight (mg)	Relative Water Content (%)
Normal	WT	27.3 ± 0.94 a	8.4 ± 0.24 a	23.3 ± 0.56 a	93.0 ± 6.8 a
	OE-3	27.1 ± 0.78 a	8.6 ± 0.31 a	22.8 ± 0.51 a	92.1 ± 4.8 a
	OE-4	26.7 ± 0.88 a	7.9 ± 0.42 a	23.4 ± 0.44 a	94.4 ± 5.5 a
	OE-6	27.4 ± 0.78 a	8.2 ± 0.32 a	23.9 ± 0.49 a	95.2 ± 4.9 a
Drought	WT	19.4 ± 0.66 b	4.9 ± 0.29 b	16.4 ± 0.51 b	40.2 ± 5.6 b
	OE-3	25.5 ± 0.84 a	7.9 ± 0.33 a	21.9 ± 0.62 a	68.6 ± 4.8 a
	OE-4	25.1 ± 0.82 a	7.8 ± 0.41 a	22.2 ± 0.53 a	66.2 ± 6.4 a
	OE-6	26.4 ± 0.77 a	8.1 ± 0.37 a	23.3 ± 0.66 a	73.5 ± 5.3 a
Heat	WT	21.8 ± 0.83 b	5.6 ± 0.28 b	19.4 ± 0.56 b	56.5 ± 4.9 b
	OE-3	27.9 ± 0.88 a	8.3 ± 0.32 a	23.5 ± 0.52 a	81.1 ± 5.1 a
	OE-4	27.3 ± 0.69 a	7.7 ± 0.42 a	23.7 ± 0.51 a	83.6 ± 6.3 a
	OE-6	26.5 ± 0.73 a	8.5 ± 0.39 a	24.5 ± 0.62 a	88.2 ± 5.2 a

Values represent means ± SE (*n* = 3). Different letters next to the numbers indicate significant difference between lines under the same conditions (*p* ≤ 0.05). WT: Wild type; SE: Standard error.

**Table 2:** Yield traits of WT and transgenic rice lines under normal and stress conditions.

Treatment	Line	Panicle Length (cm)	Number of Spikelets per Panicle	Total no. of Spikelets per Hill	Number of Filled Grains Per Hill	Filling Rate (%)	Total Grain Weight (g)
Normal	WT	17.8 ± 1.11 a	89.8 ± 8.52 a	1188.4 ± 132.3 a	1042.6 ± 141.3 a	87.3 ± 12.11 a	18.2 ± 1.32 a
	OE-3	18.2 ± 1.07 a	91.3 ± 7.61 a	1166.8 ± 143.4 a	1044.9 ± 113.5 a	89.2 ± 10.14 a	17.1 ± 1.41 a
	OE-4	18.1 ± 1.18 a	88.6 ± 9.44 a	1180.6 ± 128.7 a	1068.7 ± 131.7 a	90.2 ± 12.73 a	18.7 ± 1.36 a
	OE-6	17.8 ± 1.22 a	92.4 ± 8.66 a	1201.9 ± 144.5 a	1076.9 ± 122.8 a	89.7 ± 10.52 a	13.9 ± 1.45 a
Drought	WT	13.1 ± 1.34 b	79.7 ± 7.94 b	1020.2 ± 125.7 b	698.30 ± 133.5 b	68.1 ± 13.46 b	17.8 ± 1.41 b
	OE-3	16.9 ± 1.52 a	87.2 ± 8.14 a	1178.8 ± 112.6 a	993.7 ± 124.5 a	84.5 ± 10.32 a	17.3 ± 1.38 a
	OE-4	17.1 ± 1.44 a	88.9 ± 7.91 a	1163.9 ± 118.9 a	982.4 ± 121.5 a	84.7 ± 11.43 a	18.1 ± 1.32 a
	OE-6	17.7 ± 1.56 a	90.3 ± 8.11 a	1188.3 ± 155.3 a	1028.7 ± 118.6 a	86.6 ± 13.37 a	17.4 ± 1.31 a
Heat	WT	14.2 ± 1.39 b	81.4 ± 7.92 b	1076.9 ± 162.3 b	734.51 ± 109.2 b	68.9 ± 15.62 b	14.2 ± 1.28 b
	OE-3	17.8 ± 1.68 a	90.6 ± 8.55 a	1192.7 ± 133.5 a	982.6 ± 111.5 a	82.8 ± 16.74 a	17.9 ± 1.31 a
	OE-4	17.5 ± 1.12 a	88.5 ± 9.12 a	1203.9 ± 126.7 a	964.4 ± 108.4 a	80.6 ± 14.77 a	18.3 ± 1.28 a
	OE-6	18.3 ± 1.88 a	93.1 ± 8.32 a	1201.2 ± 152.5 a	1048.9 ± 126.8 a	87.4 ± 13.56 a	18.1 ± 1.31 a

Values represent means ± SE (*n* = 3). Different letters next to the numbers indicate significant difference between lines under the same conditions (*p* ≤ 0.05).
